# Special Issue: Natural Products: Anticancer and Beyond

**DOI:** 10.3390/molecules23061246

**Published:** 2018-05-23

**Authors:** Qingbin Cui, Dong-Hua Yang, Zhe-Sheng Chen

**Affiliations:** 1 Department of Pharmaceutical Sciences, St. John’s University, Queens, NY 11439, USA; Cuiq@stjohns.edu; 2 School of Public Health, Guangzhou Medical University, Guangzhou, Guangdong 511436, China

On the arduous and tortuous path of new drug discovery, exuberant natural products (both extracts and isolated single molecules) may serve as the shortcut or key resources of new drug candidates. Understanding of the mechanism of action and the innovative technical application in natural products research will surely be helpful in accelerating the procedure [1]. 

This special issue of *Molecules* is designed to summarize the new development and discovery of natural products as novel anticancer drug agents. We have received many article submissions for the special issue; in the end, 22 research articles were selected and published. As shown in these studies, many versatile structures exhibited anticancer effects or characteristics of overcoming drug resistance, including flavonoids, diterpenoids, triterpenoids, steroids, macrodiolides, phenolics et al., which shed light on the diversity of new drug discovery.

**Three flavonoids** were found to exhibit favorable anticancer effects. Fisetin, a flavonoid found in fruits and vegetables, has the ability to kill cancer cells. As shown in the research by Min et al., the induced apoptosis by fisetin in human renal carcinoma Caki cells was mediated through p53-dependent upregulation of death receptor 5 at the transcriptional level [2], rather than by modifying the membrane physicochemical properties [3] or inducing reactive oxygen species (ROS) as the key mechanism [2], providing an explanation of a novel mode of action of flavonoids. Wogonin, a flavonoid compound found in *Scutellaria baicalensis*, exhibited antimetastatic activities in hepatocarcinoma by inhibiting matrix metalloproteinase-9 as confirmed by the docking approach, Western blot results, and gelatin zymography analysis in the study by Hong et al. [4]. Another flavonol acylglycoside isolated from *Woodwardia unigemmata*, compound 6, showed comparable multidrug resistance (MDR) reversal effects to verapamil (a P-glycoprotein inhibitor) in doxorubicin-resistant human leukemia cell line K562/A02 cells [5], suggesting that it may serve as a sensitizing agent. 

Natural products of **terpenoids and steroids** also exhibited strong anticancer activity. As shown in the study by Chen et al., andrographolide, a labdane diterpene isolated from the leaves of *Andrographis paniculata* Nees, inhibited the proliferation of MV4-11 cells (a FLT3-positive acute myeloid leukemia cell line that displayed MDR) through the inhibition of fatty acid synthesis, iron uptake, and protein synthesis [6]. In Zhang et al.’s work, lathyrol-3-phenylacetate-5,15-diacetate (DEFL1), a lathyrane diterpenoid purified from *Euphorbia* (Caper spurge) seeds, could inhibit the proliferation of A549, KB, and HCT116 cells, among which A549 cells showed the most sensitivity. Mechanistic study showed that DEFL1 could increase ROS and decrease mitochondrial membrane potential, leading to apoptosis activated by the release of cytochrome c and activation of caspase-9 and -3 [7]. Tirucalla-8,24-diene-3β,11β-diol-7-one and eupha-8,24-diene-3β,11β-diol-7-one were new triterpenoids isolated and characterized from *Euphorbia kansui*. Both compounds exhibited moderate cytotoxicity against colon cancer HCT-116, gastric cancer MKN-45, and breast cancer MCF-7 [8]. Camelliasaponin B_1_ and camelliasaponin B_2_, triterpenoid saponins isolated and characterized from *Camellia oleifera* seeds, exhibited potent cytotoxic activity against A549, human liver tumor cells HepG2, and cervical cancer cells HeLa [9]. γ-Tocotrienol, an isoprenoid belonging to the vitamin E family, was found to induce the apoptosis of HeLa cells, accompanied by downregulation of Bcl-2, upregulation of Bax, and release of cytochrome c from mitochondria, as well as the activation of caspase-9, caspase-3, and subsequent poly (ADP-ribose) polymerase cleavage, indicating a mitochondria-mediated apoptosis mechanism [10]. Traditional Chinese medicine Chan Su is obtained from the skin and parotid venom glands of toads. Steroid arenobufagin was isolated from Chan Su and was found to promote apoptotic cell death in A549 cells through the activation of Noxa-related pathways, suggesting it to be a promising agent for patients with non-small cell lung cancer [11].

**Other different chemical-structure-related products** also possessed specific anticancer effects. Elaiophylin, a 16-member macrodiolide antibiotic extracted from *Streptomyces melanosporus*, exhibited potent antiangiogenic activity in vitro and in vivo at nontoxic concentrations in angiogenesis assays as shown in the study by Lim et al. Elaiophylin could inhibit hypoxia-inducible factor-1α and vascular endothelial growth factor (VEGF) receptor 2, leading to the inhibition of VEGF-induced proliferation, migration, adhesion, invasion, and tube formation of human umbilical vein endothelial cells (HUVECs) [12]. Compound (E)-caffeic acid-9-*O*-β-d-xylpyranosyl-(1,2)-β-d-glucopyranosyl ester, a phenolic isolated and identified from *Dryopteris fragrans* (L.), showed potent anticancer effects on A549 cells, breast cancer cells MCF-7, and gastric cancer cells SGC7901 and HUVECs, among which MCF-7 was the most sensitive [13]. 

**Natural product extracts** also shine in cancer treatment and are widely used, especially in Asia. In Seifaddinipour et al.’s study, ethyl acetate extracts from pistachio (*Pistacia vera* L.) hulls, abundant with phenolic and flavonoid compounds, showed potent apoptosis-inducing and angiogenesis inhibition effects on MCF-7 cells, human colon cancer HT-29, and HCT-116 cells [14]. Essential oils (a concentrated hydrophobic liquid containing volatile aroma compounds) from *Origanum vulgare* subsp *hirtum*, had a lower IC_50_ in hepatocarcinoma HepG2 cells than in the healthy human renal cells HEK293, indicating its selectivity to be utilized as cancer therapeutic agents [15]. Combination of *Prunus spinosa* Trigno ecotype (PsT) drupe extract with a nutraceutical activator complex (NAC) made of amino acids, vitamins, and mineral salt blends, which was named PsT + NAC^®^, showed anticancer activity in colon carcinoma cells HCT116 and SW480 cells via apoptosis-induction; by contrast, it did not alter normal cells [16]. *Vernonia amygdalina* Delile methanolic extracts exhibited significant growth-inhibitory effects on human androgen-independent prostate cancer cells PC-3 through the induction of cell growth arrest, DNA damage, apoptosis, and necrosis, demonstrating novel possibilities for developing prostate cancer therapies [17]. In Qiao et al.’s study, the polyyne-enriched extract from *O. elatus* (PEO) significantly improved body weight changes and reduced the tumor burden and tumor multiplicity in test mice compared with the untreated *Apc*^Min/+^ mice. Further study indicated that PEO could reduce the expression of β-catenin and cyclinD1 in both small intestine and colon tissues [18]. As shown in Moreno-Celis et al.’s work, tepary bean lectin fraction (TBLF) significantly decreased early tumorigenesis triggered by 1,2-dimethylhydrazine by 70% in rat models. TBLF exhibited antiproliferative and proapoptotic effects in the azoxy-methane/dextran sodium sulfate-induced in vivo model through decreasing the signal transduction pathway protein Akt in its activated form and increasing the activity of caspase-3 [19]. Finally, a review by Reddy et al. described the chemical compounds of *Angelica gigas* Nakai and their biosynthesis, efficient extraction and formulation methods, and underlying mechanisms for chronic diseases such as cervical cancer, prostate cancer, melanoma, bladder and colon cancer, lung cancer, and sarcoma were summarized, providing useful information for researchers in this area [20].

Most natural products fail to enter clinical application due to poor pharmacokinetic profiles. Two studies related to **structural modifications** of natural products are included in this special issue. Gambogic acid, a xanthonoid derived from *Garcinia*, possessed poor aqueous solubility and cell permeability. DDO-6318, with a 1,2,3-triazole ring scaffold, was a synthetic analogue of gambogic acid guided by click chemistry. This new compound showed better drug properties as well as in vivo anticancer activity [21]. An analogue with better bioavailability modified based on curcumin (the principal curcuminoid of turmeric [*Curcuma longa*]), DK1, was found to exhibit cytotoxic effects on U-2OS and MG-63 osteosarcoma cell lines by inducing the expression of caspase-3, caspase-9, and Bax, activating apoptosis through a mitochondria-dependent signaling pathway [22]. These two research studies provide a novel strategy for further structural modification of natural products. 

Several research studies were also conducted to overcome drug-resistant cancers in this special issue. Examples include compound 6 [5] and andrographolide [6]. 6-gingerol, 10-gingerol, 6-shogaol, and 10-shogaol, isolated from ginger, significantly inhibited the proliferation of docetaxel-resistant human prostate cancer cell line PC3R via downregulation of multidrug resistance-associated protein 1 and glutathione-S-transferase protein expression [23]. N-Butylidenephthalide, an active component of *Radix Angelica Sinensis (danggui)*, showed potent inhibition and radiosensitization in human MDA-MB-231 and MCF-7 breast cancer cell lines [24]. These studies indicated that many natural products may work as sensitizing agents for MDR cancer treatment. 

In conclusion, this special issue provides multiple research topics on the potential anticancer characteristics of natural products and some of their synthetic analogues. Many of them demonstrated certain MDR-overcoming effects in cancer cells that warrant further research. The insights of these studies will shed light on new drug discoveries and contribute to novel anticancer therapeutics. 
